# Ethical and Clinical Considerations in the Workup of HIV-Associated Neurocognitive Decline

**DOI:** 10.7759/cureus.97348

**Published:** 2025-11-20

**Authors:** Kayla Davis, Krista-Gaie Grant, John M Sousou, Sunday Ilechukwu

**Affiliations:** 1 Department of Psychiatry, University of Florida College of Medicine, Gainesville, USA; 2 Department of Internal Medicine, University of Florida College of Medicine - Jacksonville, Jacksonville, USA

**Keywords:** bioethics, end-of-life care, hiv-associated dementia, hiv-associated neurocognitive disorder, hiv encephalitis, lgbt health, neurocognitive decline, progressive multifocal leukoencephalopathy, stigma, surrogate decision-making

## Abstract

Human immunodeficiency virus (HIV) can invade the central nervous system, leading to a spectrum of neurocognitive disorders known as HIV-associated neurocognitive disorder (HAND). Despite the widespread use of combination antiretroviral therapy (cART), patients remain at risk for HAND and other neurologic complications, including progressive multifocal leukoencephalopathy (PML) and HIV encephalitis (HIVE). Differentiating among these conditions is crucial, given their distinct etiologies, prognoses, and management strategies.

We describe a 37-year-old man with a six-month history of untreated HIV who presented with a four-week history of progressive cognitive decline, confusion, incontinence, and loss of functional independence. T2/fluid-attenuated inversion recovery (FLAIR) MRI revealed bilateral cerebral white matter hyperintensity with patchy involvement of the cerebellar peduncles, excluding U-fibers and the corpus callosum, raising concern for PML, HIVE, or advanced HAND. His clinical course was complicated by inconsistent treatment adherence, medication refusal, and eventual determination of decision-making incapacity. Family conflict related to stigma rooted in his bisexual identity and HIV status contributed to delays in timely diagnostic testing and transition to hospice care. These delays were partly a result of his decision-making surrogate's difficulty in communicating prompt healthcare decisions due to family discord.

This case highlights the dual challenges of clinical ambiguity and ethical conflict in the care of patients with advanced HIV. Diagnostic evaluation of HIV-associated cognitive decline requires integration of neuroimaging, cerebrospinal fluid studies, and neuropsychological testing. However, even when clinical pathways are evident, stigma, fractured family dynamics, and the absence of advance directives can obstruct timely and appropriate care.

Optimal management of HIV-associated neurocognitive decline extends beyond biomedical treatment. A stigma-informed, ethically sensitive approach, including early counseling, advance care planning, surrogate support, and ethics consultation, is essential to protect patient autonomy and improve healthcare outcomes. This case emphasizes the need to address psychosocial and ethical barriers alongside clinical management in patients with severe HIV-related neurocognitive impairment.

## Introduction

Human immunodeficiency virus (HIV) can traverse the blood-brain barrier and injure the central nervous system, producing a spectrum of neuropsychiatric complications collectively termed HIV-associated neurocognitive disorder (HAND) [1]. HAND comprises asymptomatic neurocognitive impairment (ANI), mild neurocognitive disorder (MND), and HIV-associated dementia (HAD). HAD, which was formerly called AIDS dementia complex (ADC), was highly prevalent before combination antiretroviral therapy (ART) became widely available. The Frascati classification is a three-tier system developed by the United States National Institutes of Health in 2007 to classify the severity of HIV associated neurocognitive decline [2]. The three tiers range from ANI to dementia that is solely attributable to HIV [2]. Since the mid-1990s, ART has reduced the incidence of HIV-related dementia; patients now more often present with milder phenotypes, reflected in the Frascati classification [2]. Importantly, even with systemic viral suppression, HIV can persist in the CNS (e.g., as a reservoir or with CSF viral “escape”), and the term HIV encephalopathy (HIVE) is sometimes used interchangeably with HAD to denote severe neurocognitive decline [2].

HIV is also associated with other neurologic disorders, notably progressive multifocal leukoencephalopathy (PML), HIVE, and leukodystrophies [3]. PML is a subacute-onset, rapidly progressive, often fatal demyelinating disease caused by JC virus reactivation in the setting of cellular immunosuppression. It typically affects subcortical white matter and presents with variable focal deficits such as gait disturbance, weakness, cognitive decline, sensory loss, or visual changes [3]. In contrast, HIVE arises from direct HIV-mediated neuroinflammation with macrophage/microglial activation and synaptodendritic injury; histopathology can show perivascular macrophages, multinucleated giant cells, necrotizing microglial nodules, and white-matter rarefaction, accompanied clinically by progressive memory impairment, motor dysfunction, and behavioral change [4]. Leukodystrophies contribute to neurocognitive decline through genetically mediated white-matter degeneration and may confound the differential in selected patients [5].

Distinguishing among HAND, PML, and HIVE is essential because the differential diagnosis guides both workup and treatment. For example, progression along the HAND spectrum can be slowed with effective ART, whereas PML and HIVE may present with overlapping symptoms but require different diagnostic strategies (e.g., CSF JC virus polymerase chain reaction (PCR) for PML) and carry distinct prognostic implications [1-5]. Timely recognition is crucial to prevent further neurologic injury and functional decline. These cases also raise ethical considerations: cognitive impairment can compromise autonomy and necessitate a surrogate decision-maker; when no surrogate is pre-designated, or when stigma and family conflict complicate choices, decision-making may be delayed and care outcomes adversely affected.

## Case presentation

The patient was a 37-year-old HIV-positive male admitted for physical decline and passive suicidal ideation. The patient verbally expressed suicidal thoughts but denied intent or a plan. He presented with confusion, historical inconsistencies, incontinence, and inability to perform activities of daily living (ADLs). He had been diagnosed with HIV months earlier but declined treatment. Admission laboratories are summarized in Table 1 and were notable for anemia (hemoglobin 8.9 g/dL, hematocrit 27.5%), hypoalbuminemia (2.6 g/dL), mild hypocalcemia (8.0 mg/dL), and vitamin D deficiency (17.3 ng/mL). Electrolytes showed mild hypokalemia (3.4 mmol/L) and mildly decreased bicarbonate (21 mmol/L). CD4 count was 78 cells/µL. Infectious workup revealed *Candida glabrata* in bronchoalveolar lavage (BAL) fluid, while *Aspergillus* antigen, acid-fast bacilli (AFB) cultures, *Legionella* antigen, and blood cultures were negative; QuantiFERON-TB was indeterminate; syphilis serology was nonreactive. Urine drug screen was positive for benzodiazepines.

**Table 1:** Laboratory investigations obtained on hospital admission, including serum chemistry, liver function tests, complete blood count, immunologic studies, and microbiologic testing. Abnormal values are indicated as high (H) or low (L) relative to reference ranges. eGFR: estimated glomerular filtration rate; AST: aspartate transaminase; ALT: alanine transaminase; MCV: mean corpuscular volume; MCH: mean corpuscular volume; MCHC: mean corpuscular hemoglobin; RDW: red cell distribution width; RDW-SD: red cell distribution width - standard deviation; MPV: mean platelet volume; BAL: bronchoalveolar lavage; AFB: acid-fast bacilli; PCR: polymerase chain reaction

Parameter	Patient Value	Reference Range
Basic Metabolic Panel		
Glucose	81 mg/dL	65-99 mg/dL
Blood Urea Nitrogen (BUN)	13 mg/dL	9-20 mg/dL
Creatinine	0.7 mg/dL	0.5-1.2 mg/dL
eGFR (2021 CKD-EPI)	>90 mL/min	-
Sodium	138 mmol/L	135-145 mmol/L
Potassium	3.4 mmol/L (L)	3.5-5.0 mmol/L
Chloride	105 mmol/L	98-108 mmol/L
CO_2_ (Bicarbonate)	21 mmol/L (L)	23-32 mmol/L
Anion Gap	12 mmol/L	5-15 mmol/L
Calcium	8.0 mg/dL (L)	8.4-10.5 mg/dL
Phosphate (PO_4_)	4.1 mg/dL	2.5-4.6 mg/dL
Magnesium	2.0 mg/dL	1.7-2.5 mg/dL
Liver Function Tests		
Total Protein	7.3 g/dL	6.0-8.2 g/dL
Albumin	2.6 g/dL (L)	3.5-5.0 g/dL
Total Bilirubin	0.2 mg/dL	0.0-1.3 mg/dL
Alkaline Phosphatase	88 U/L	0-125 U/L
AST	26 U/L	0-45 U/L
ALT	31 U/L	0-40 U/L
Complete Blood Count		
WBC	4.71 k/µL	4.6-10.8 k/µL
RBC	2.79 M/µL (L)	4.44-6.1 M/µL
Hemoglobin	8.9 g/dL (L)	13.9-18.0 g/dL
Hematocrit	27.5% (L)	41.0-52.0%
MCV	98.6 fL (H)	80-98 fL
MCH	31.9 pg	27-33.3 pg
MCHC	32.4 g/dL	31.8-37.1 g/dL
Platelets	322 k/µL	130-440 k/µL
RDW	14.7% (H)	11.5-14.5%
RDW-SD	53.2 fL (H)	39.0-52.2 fL
MPV	8.9 fL	7.4-10.5 fL
Neutrophils	54.80%	54-65%
Neutrophils (Abs)	2.58 k/µL	1.8-7.8 k/µL
Lymphocytes	25.90%	25-33%
Lymphocytes (Abs)	1.22 k/µL	1.2-3.6 k/µL
Monocytes	16.6% (H)	3-7%
Monocytes (Abs)	0.78 k/µL (H)	0.14-0.76 k/µL
Eosinophils	0.60%	0-3%
Basophils	0.60%	0-2%
Immature Granulocytes	1.50%	0-2%
Additional Labs		
CD4 Count	78 cells/µL (L)	500-1500 cells/µL
Vitamin B12	>2000 pg/mL (H)	200-900 pg/mL
Vitamin D (25-OH)	17.3 ng/mL (L)	30-100 ng/mL
Legionella Urinary Ag	Negative	Negative
BAL Culture (Fungus)	*Candida glabrata* (few)	-
BAL AFB Smear/Culture	Negative	Negative
Aspergillus Ag (BAL)	Not Detected	<0.50 Index Value
Chlamydia PCR	Not Detected	Not Detected
Neisseria PCR	Not Detected	Not Detected
Hepatitis B Surface Ab	Negative	-
QuantiFERON TB	Indeterminate	Negative
Blood Cultures	No Growth (five days)	No Growth

**Figure 1:**
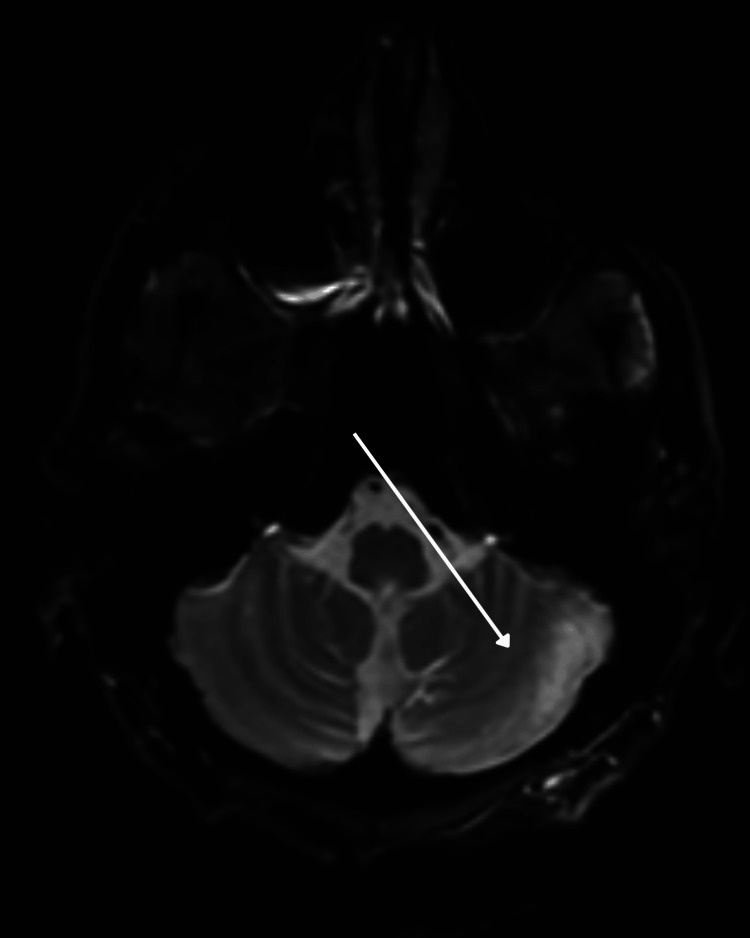
Axial T2/FLAIR MRI showing extensive bilateral cerebral white matter hyperintensity with patchy involvement of the anterior and middle cerebellar peduncles, more prominent on the right (arrow). FLAIR: fluid-attenuated inversion recovery

**Figure 2:**
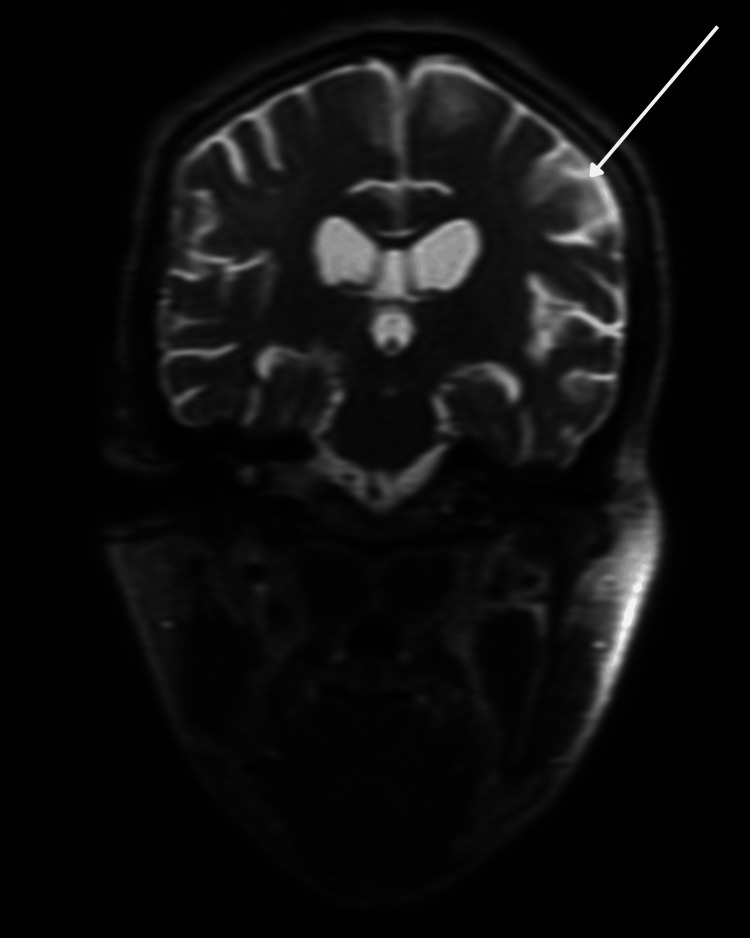
Coronal T2/FLAIR MRI demonstrating diffuse symmetric periventricular and deep white matter hyperintensity with subtle involvement of the bilateral gangliocapsular regions (arrow). FLAIR: fluid-attenuated inversion recovery

Neurologic examination revealed impaired attention with right-sided hemineglect on bedside testing; language was fluent. MRI demonstrated extensive bilateral white-matter T2/fluid-attenuated inversion recovery (FLAIR) abnormalities, with patchy hyperintensity in the right > left anterior and middle cerebellar peduncles and mild atrophy, excluding U-fibers and the corpus callosum, and without definite enhancement. These findings are shown in Figure 1 (axial T2/FLAIR) and Figure 2 (coronal T2/FLAIR) and raised concern for PML while HIVE and advanced HAND/HAD remained in the differential [3-5].

In the interview, the patient’s father assisted with communication. Records documented a 10-day hospitalization two months earlier for psychosis, during which he was diagnosed with HIVE and noted to have severe neurocognitive decline with a limited prognosis. At that time, his father requested caregiver resources, and the patient was discharged to hospice. He did not transition as planned and was subsequently readmitted.

During the current admission, he demonstrated persistent treatment refusal behaviors, prompting a formal capacity evaluation, and was ultimately deemed incapacitated. His father became the healthcare proxy, and goals-of-care (GOC) discussions focused on whether to pursue further diagnostic evaluation (neuropsychological testing and lumbar puncture with CSF studies) or to prioritize long-term hospice for comfort. The father expressed a desire to “give 30 days in the hospital,” but could not articulate a clinical rationale. He later disclosed family discord affecting decision-making: after the HIV diagnosis, the patient, who was bisexual, had confided in him. The father reported the patient’s partners to the health department, which strained relations with the extended family, who believed this exposed the patient’s sexuality and HIV status. Due to stigma surrounding bisexuality and HIV disclosure, family conflict delayed the father’s ability to finalize treatment decisions, which in turn hindered the team’s ability to complete the work-up, initiate sustained disease-directed therapy, or transition promptly to comfort-focused care.

Treatment, response, and follow-up

The patient was started on ART with bictegravir/emtricitabine/tenofovir alafenamide (Biktarvy) and atovaquone for *Pneumocystis jirovecii *prophylaxis; adherence was poor. On admission, infectious diseases and pulmonary services recommended empiric therapy for community-acquired pneumonia with a five-day course of azithromycin and ceftriaxone. Bronchoscopy with BAL revealed *C. glabrata*; he received fluconazole for oral and probable esophageal candidiasis. Supportive therapies included pantoprazole for gastroesophageal reflux disease (GERD), ergocalciferol for vitamin D deficiency, and wound care for a stage III sacral pressure injury and a posterior scrotal wound.

Symptom-directed palliative measures included acetaminophen for fever/pain, ondansetron for nausea, laxatives for constipation, glycopyrrolate for terminal secretions, and haloperidol or lorazepam for anxiety, agitation, or delirium. Oxycodone was used as needed for pain control, and olanzapine was initiated for agitation with dose titration during hospitalization. Nutritional support addressed moderate protein-calorie malnutrition; despite interventions, he remained cachectic (BMI 17).

Despite medical management, the patient’s course was complicated by HIVE, advanced immunosuppression (CD4 78 cells/µL; HIV RNA >770,000 copies/mL), malnutrition, and multiple comorbid infections. Functional and nutritional status continued to decline. After further GOC discussions, the family elected a comfort-focused approach; ART and atovaquone were discontinued to reduce pill burden. He was transferred to an inpatient hospice unit for end-of-life care. Overall, the findings are clinically most consistent with PML, with advanced HAND/HIVE remaining in the differential. CSF JCV PCR was not obtained because of initial refusal, subsequent loss of decision-making capacity, and unresolved surrogate conflict [3-6].

## Discussion

Differential diagnosis

Although HAND, HIVE, PML, and leukodystrophy can all present with cognitive decline in individuals with HIV, differences in symptomatology, imaging, and diagnostic testing assist in distinguishing among them.

HAND

HAND typically presents gradually with cognitive slowing, impaired concentration/attention, and memory deficits [1]. In advanced stages (HAD), a subcortical motor pattern emerges (slowed gait, reduced finger tapping/dexterity) and may progress to mutism and incontinence [1]. HAND is more common in patients with low CD4 counts or those not receiving ART [1]. Imaging can show diffuse or patchy T2/FLAIR white-matter change with global or frontal-subcortical atrophy, rather than a single focal pattern [1,2].

HIVE

HIVE reflects HIV-mediated neuroinflammation (macrophage/microglial activation with synaptodendritic injury) [4]. Symptoms include psychomotor slowing, impaired attention/memory, and executive dysfunction; cortical language/gnosis is relatively preserved, and gait ataxia or tremor may occur [4]. The overall course is progressive (day-to-day fluctuation can happen), and MRI commonly shows diffuse symmetric white-matter abnormality with atrophy; imaging is supportive rather than diagnostic [4].

PML

PML from JC virus reactivation typically follows a subacute, steadily progressive course (weeks to months) with focal deficits such as gait disturbance, weakness, cognitive changes, sensory symptoms, or visual field deficits [3]. MRI usually demonstrates asymmetric, non-enhancing T2/FLAIR hyperintense and T1 hypointense white-matter lesions with little mass effect [3]. CSF JCV PCR is often positive but not uniformly so; early false negatives occur, and repeat testing can be appropriate when clinical suspicion remains high [3]. Histopathology shows multifocal demyelination, atypical oligodendrocytes, and enlarged astrocytes [3]. Despite supportive clinical evidence, a definitive diagnosis of PML cannot be established without a positive CSF JCV PCR result [3].

Leukodystrophy

Genetic leukoencephalopathies can mimic infectious/inflammatory white-matter disease; age of onset and molecular testing guide diagnosis [5,7].

The patient’s subacute decline with right-sided hemineglect, plus MRI showing confluent bilateral white-matter hyperintensities with right-predominant middle cerebellar peduncle involvement and no definite enhancement, is most consistent with PML, with advanced HAND/HIVE remaining in the differential given diffuse disease and profound immunosuppression [3-5,8]. Diagnostic certainty is limited by the absence of CSF; JCV PCR (and repeat testing if initially negative) would have been the next step, but was not obtained due to refusal, subsequent incapacity, and unresolved surrogate conflict [3]. MRI assists in pattern recognition and the exclusion of alternative diagnoses (see Table 2).

**Table 2:** Comparison of clinical, radiologic, and pathologic features of PML, HAND, and HIV encephalopathy PML: progressive multifocal leukoencephalopathy; HIV: human immunodeficiency virus; HAND: HIV-associated neurocognitive disorder; CSF: cerebrospinal fluid; PCR: polymerase chain reaction; JC virus: John Cunningham virus; FLAIR: fluid-attenuated inversion recovery; cART: combination antiretroviral therapy; ANI: asymptomatic neurocognitive impairment; MND: mild neurocognitive disorder; HAD: HIV-associated dementia; ART: antiretroviral therapy; HIVE: HIV encephalitis Credit: Table created by the authors.

Features	PML	HAND	HIVE
Etiology	JC virus reactivation in an immunocompromised host	HIV replication in the CNS	HIV-induced inflammation and neuronal damage
Onset and Progression	Subacute onset with rapid progression	Gradual onset, chronic progression	Waxing/waning day-to-day but overall progressive; can occur with CD4 >350
Key Symptoms	Gait disturbance, visual field deficits, cognitive decline	Cognitive slowing, motor impairment, and memory deficits	Psychomotor slowing, memory and attention deficits, intact cortical function; gait ataxia, tremor
MRI Findings	Asymmetric white matter lesions, T2/FLAIR hyperintense, T1 hypointense	Subcortical atrophy, ventricular enlargement	Symmetric subcortical T2 hyperintensity; T1 variable (reports of hyperintensity exist); cerebral atrophy
CSF PCR	JCV PCR often positive (≈70-95% depending on assay/setting); if negative with high suspicion, repeat	Usually negative, may show elevated HIV viral load	Negative
Histopathology	Multifocal demyelination, enlarged astrocytes, and oligodendrocytes	Not typically diagnostic via histology	Perivascular macrophages, multinucleated giant cells, and white matter thinning
Diagnostic Criteria	No formal criteria; diagnosis based on MRI and CSF	Frascati criteria (ANI, MND, HAD)	No specific criteria; clinical and radiologic diagnosis
Treatment	Initiate or optimize cART	ART is the mainstay of treatment	ART is the mainstay; fatal within one year without treatment

Bioethical considerations

Although there were clear diagnostic criteria for the patient’s workup, significant bioethical considerations arose in his care. The principles of nonmaleficence (do no harm) and beneficence (acting in the patient’s best interest while maximizing quality of life) are central to the physician’s role. Barriers to upholding these principles were evident. Family discord and delays in decision-making resulted in the postponement of diagnostic tests such as lumbar puncture, as well as delays in establishing a definitive treatment plan. Given advanced neurocognitive decline and poor prognosis, discussions regarding aggressive interventions versus hospice care were initiated. However, because the patient was incapacitated and lacked an advance directive, his wishes were unknown. Although his father was the designated healthcare surrogate, he hesitated to make decisions without broader family input, further delaying care. This created challenges to respecting the patient’s autonomy, as neither the patient nor the family effectively communicated his preferences to the care team.

A central source of family discord was stigma surrounding the patient’s sexuality. While the father acknowledged the patient’s bisexuality, other relatives insisted he was heterosexual and resented the father’s disclosure. Such stigma likely influenced family dynamics, delayed decision-making, and may also have contributed to the patient’s earlier non-adherence to ART [9]. Prior research demonstrates that both internalized and societal stigma regarding sexuality and HIV diagnosis are associated with decreased treatment adherence. This case raises important considerations regarding the adequacy of counseling provided to the patient about the risks associated with declining ART, including the potential for severe neurocognitive decline. It also emphasizes the importance of offering supportive resources to address stigma and misinformation surrounding HIV and sexual identity. Additionally, the case illustrates how earlier counseling and the establishment of an advance directive might have influenced the patient’s clinical course and end-of-life decision-making. These considerations highlight the need to safeguard informed consent, autonomy, and beneficence in the care of patients with HIV and neurocognitive impairment.

Stigma’s role in delayed diagnosis and management decisions

HIV-related stigma played a central role in this patient’s delayed diagnosis and inconsistent adherence to ART. Stigma is strongly associated with late presentation to care: a meta-analysis found that patients who perceived high HIV-related stigma were twice as likely to present late for treatment compared with those reporting low stigma [10]. Among gay and bisexual men, stigma contributes to avoidance or delay of HIV testing [11]. Fear of discrimination and negative community attitudes likely shaped this patient’s reluctance to test early and engage in care. Once diagnosed, his intermittent adherence is also explained by internalized stigma. Individuals who conceal their HIV status as a coping strategy often struggle with medication consistency.

Stigma surrounding both the patient’s HIV status and bisexual identity exacerbated family conflict. Parental rejection of sexual minority identity is associated with adverse health outcomes [12]. In this case, the family’s difficulty accepting his orientation delayed unified support for treatment decisions. Earlier initiation of ART at higher CD4 counts reduces HAND severity by limiting CNS inflammation and T-cell trafficking [13]. It is likely the patient’s cognitive decline would have been less pronounced had ART been initiated earlier.

Evidence-based approaches exist to reduce HIV-related stigma at multiple levels. Family- and community-level interventions, such as open education and stigma-reduction activities, can foster supportive attitudes [14]. Media-based efforts, including radio campaigns, have reduced stigma in affected communities [15]. In healthcare settings, provider-focused strategies such as cultural-sensitivity training, implicit-bias education, and ongoing assessments of staff knowledge and attitudes decrease stigma and improve patient interactions [16]. Community outreach and peer support groups help humanize HIV, reduce isolation, and strengthen adherence [17]. Together, these multilevel interventions address stigma, promote adherence, and ultimately improve clinical outcomes and quality of life for persons living with HIV.

Family dynamics and decision-making conflicts

This case unfolded within a complex family context marked by stigma and mistrust. The patient’s bisexual identity and HIV diagnosis strained family relationships, and conflict intensified after the father disclosed his son’s HIV status to local health authorities so that partners could be notified. Although well-intentioned, the disclosure highlighted the ethical tension between confidentiality and perceived public-health duty. In general, HIV status is considered confidential, with disclosure permissible only under limited circumstances, such as when needed for surrogate decision-making or to prevent direct risk to others [18].

Family discord directly undermined surrogate decision-making. By default, the patient’s father was his healthcare proxy once the patient lost capacity. The patient's decision-making capacity was assessed using a set of standardized prompts and was confirmed by consensus among the primary medical, neurological, and psychiatric teams. However, effective surrogate decision-making requires both legal authority and an ability to represent the patient’s values free of conflicting motives. An appropriate surrogate must act in the patient’s best interest, not under pressure from family disputes. In this case, the father’s judgment was clouded by fear, stigma, and ongoing conflict, leaving him paralyzed by competing demands. Rather than focusing on his son’s likely preferences, such as whether to continue neurological evaluation or pursue comfort care, he deferred to divided relatives, leading to indecision and inconsistency.

Several strategies may mitigate surrogate-family conflict. Mediation by a neutral party can facilitate structured dialogue, reduce tensions, and promote consensus around patient-centered goals. Ethics consultation provides an impartial framework to analyze values, obligations, and ethical principles, ensuring the patient’s perspective is represented. Despite their utility, ethics services remain underutilized: one survey found few facilities routinely employ ethics consultants in family conflicts, even though 71% of physicians reported encountering such disputes [19]. Finally, clarifying decision-making authority is essential. While surrogates may consider input from others, they are not obligated to reach a unanimous agreement and should prioritize the patient’s known or likely wishes above family pressures.

Recommendations and future directions

This case emphasizes the necessity of a proactive, multidisciplinary approach when managing patients with HAD complicated by psychosocial conflict. Several measures can improve outcomes in similar situations.

First, early ethics involvement should be standard when surrogate-family discord emerges or when a patient’s wishes are unclear. Protocols that trigger automatic ethics consultation can prevent care delays and help surrogates understand their role, namely, to prioritize the patient’s values over family politics [19]. Ethics committees also provide structured frameworks to resolve conflicts and ensure decisions remain patient-centered.

Second, the initial disclosure of an HIV diagnosis must be handled with empathy, structure, and immediate linkage to support. Delivering written and verbal information, addressing stigma directly, and connecting patients and families to counseling or case management can normalize HIV as a chronic, manageable condition. In this case, structured counseling at diagnosis might have reduced the shock and stigma that later undermined care. Ongoing psychosocial support, particularly counseling for both patients and families, should be integrated into HIV care to address internalized stigma, improve ART adherence, and set realistic expectations about potential neurocognitive complications [17].

Third, healthcare institutions must invest in LGBTQ+ cultural competence. Training in implicit bias, respectful communication, and the specific needs of LGBTQ+ patients should be mandatory for all clinical and support staff. Research shows such interventions improve treatment adherence and mental-health outcomes [17]. Hospitals should reinforce inclusivity through non-discrimination policies, visible affirming signage, support groups, and partnerships with community organizations to combat stigma beyond clinical settings.

Ultimately, this case demonstrates that waiting until conflicts erupt is too late. Ethical guidance, structured communication, psychosocial support, and institutional stigma reduction must be built into HIV care from the start. These measures are both preventative and restorative, ensuring patients and families are informed, supported, and respected throughout the illness trajectory.

## Conclusions

This case illustrates how clinical outcomes in advanced HIV are inseparable from the ethical and social context of care. Stigma and family conflict delayed ART initiation and adherence, and those delays contributed to irreversible neurocognitive decline. Decision-making was further compromised when surrogate authority collided with fractured family dynamics and bias, impeding timely advocacy for the patient’s best interests. The lesson is plain: even sound medical plans can be undermined by unaddressed psychosocial barriers. Improved care requires a stigma-informed model embedded in everyday practice, not added after problems arise. Practical elements include routine screening for stigma-related stress at intake and at inflection points; staff training in clear, compassionate communication; and early linkage to peer mentorship or community supports. Bringing stigma into the clinical conversation normalizes concerns before they derail testing, treatment, or GOC discussions. A truly holistic approach treats psychosocial and ethical obstacles, denial, mistrust, fear of discrimination, and family discord with the same urgency as biomedical complications.

For patients with advanced HIV and neurocognitive impairment, care must extend beyond prescribing ART or treating opportunistic infections. It must include explicit education, empathy, and structured support for surrogates, alongside coordinated, patient-centered planning. In similar cases, a parallel pathway is essential: probability-based diagnostics (acknowledging when testing is infeasible), moving alongside early ethics involvement, clear time-limited trials with objective milestones, and proactive advance-care planning. Through stigma-informed and ethically attuned care, clinicians can better safeguard the dignity and autonomy of people living with HIV, even in the most complex circumstances.
